# The diagnosis and management of rare cystic liver metastases from nasopharyngeal carcinoma: A case report

**DOI:** 10.1097/MD.0000000000011257

**Published:** 2018-06-29

**Authors:** Qiuxia Liu, Jianfang Wang, Caiping Sun, Jun Xu

**Affiliations:** Department of Oncology, Chemotherapy and Radiotherapy, Shaoxing People's Hospital, Shaoxing Hospital of Zhejiang University, Zhejiang, Shaoxing 312000, China.

**Keywords:** cystic liver metastases, liver cysts, liver resection, nasopharyngeal carcinoma, TACE

## Abstract

**Rational:**

Nasopharyngeal carcinoma (NPC) with cystic liver metastases is so rarely observed that there are only three cases reported in the published literature.

**Patient concerns:**

We present a case of NPC that received complete response after chemotherapy and definitive radiotherapy, but a liver cystic lesion was revealed on abdominal sonogram three months after the initial therapy. The cystic liver lesion initially resembled a simple liver cyst with fast growth, and then evolved into an abscess-like mass after a short term. Though abscess drainage was performed, and the mass shrank significantly, but it returned to previous size two months later.

**Diagnoses:**

Surgical resection was administrated both for diagnosis and treatment, and eventually the lesion was histologically demonstrated to be a liver metastasis. Eight months after the partial hepatectomy, cystic liver metastases recurred on computed tomography (CT) scan.

**Interventions:**

Though palliative systematic chemotherapy including paclitaxel, cisplatin, gemcitabine, navobine and anti-epidemal growth factor receptor (anti-EGFR) molecular-targeted therapy were performed, the cystic metastases still gradually progressed. Then Transcatheter Hepatic Artery Chemoembolization (TACE) was administrated for five times, and all the lesions were obviously decreased in size.

**Outcomes:**

After TACE treatment, the liver metastases maintained stable for six months, but lung metastases were noted. Finally, the patient died of liver failure.

**Lessons:**

The rare cystic appearance may be a special form which exists for liver metastases of NPC, indicating poor prognosis. Oncologists need to enhance the recognition and diagnosis level of this type of metastases. Intense follow-up and early diagnosis are important. While emphasizing the importance of local therapy and personal principles for liver metastases, TACE may be a preferred method for unresectable cystic liver metastases from NPC.

## 1 Introduction

Nasopharyngeal carcinoma (NPC) is an epidemic in south-east Asia, especially in southern China, where nonkeratinized undifferentiated subtype accounts for approximately 95% of NPC cases and the propensity to distant metastasize is the greatest, ranging from 17% to 54%.^[1]^ Although the excellent local control has been achieved and the overall progress has improved significantly in recent years due to the intensity modulated radiotherapy (IMRT) technology and the application of concomitant chemotherapy, exceeding 90% at 2 to 5 years,^[2]^ distant failure remains a major challenge in the management of NPC. Liver is the third most frequent metastatic site, and liver metastases usually present as solitary or multiple solid masses on imaging, while cystic appearance is extremely rare.

We report a patient with NPC who presented a single cystic liver cyst at her first follow-up, the lesion evolved into an abscess-like mass afterward, then was histologically demonstrated to be a liver metastasis from NPC 7 months later. Ethics approval was gained from the Shaoxing People's Hospital Ethics Committee and the patient's consent was also obtained.

## 2 Case report

A 40-year-old female, with 3-month history of nasal obstruction and tinnitus was admitted in August 2012. Nasopharyngeal endoscopy and biopsy already had been performed in another hospital, showing nonkeratinizing undifferentiated NPC. This histopathologic diagnosis was confirmed in our center. Magnetic resonance imaging (MRI) of the nasopharynx and neck revealed the tumor was confined to the nasopharynx and the bilateral locoregional cervical lymph nodes enlarged with its greatest dimension of 2 cm. Chest computed tomography (CT) scan, ultrasound of abdomen, and whole-body bone scan ruled out distant metastases. So clinical staging was determined to be T1N2M0, IIIA according to American Joint Committee on Cancer TNM Staging System for NPC (7th ed, 2010).

The patient was treated with definitive IMRT to 7050 cGy for primary tumor and 6600 cGy for infiltrated regional lymph nodes. Concurrent chemotherapy based on cisplatin and 5-flurorouracil was administrated for 2 cycles and then 2-cycle chemotherapy was given subsequently to consolidate the efficiency with the same regimen. At the end of therapy, she obtained clinical complete response by nasopharynx and neck MRI.

**Figure 1:**
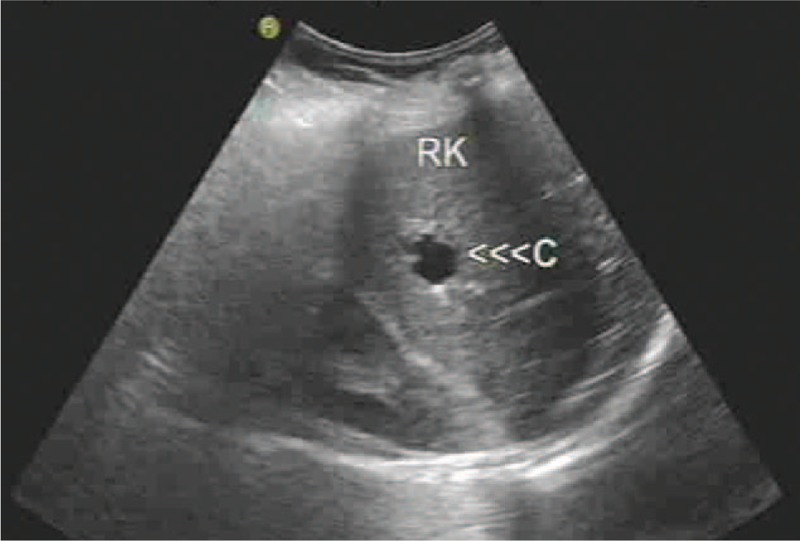
Abdominal sonogram revealing a liver cystic lesion with thin wall and smooth margin in the right liver lobe.

**Figure 2:**
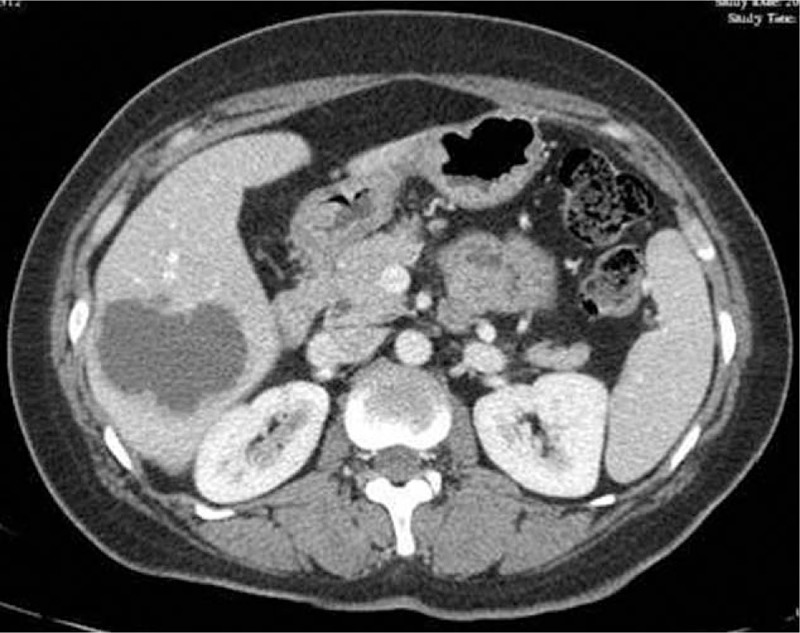
Computed tomography presenting a low density and heterogeneous lesion taking irregular wall and incompletely septa with strong contrast enhancement.

**Figure 3:**
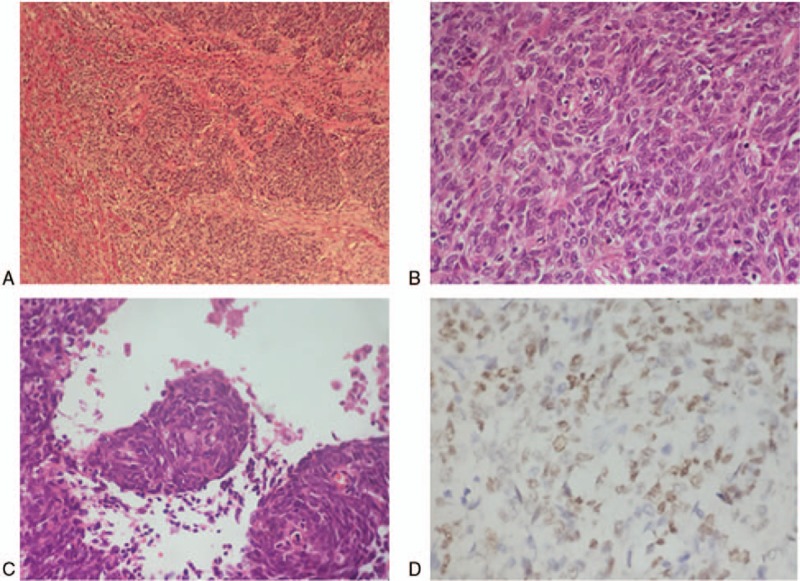
Resected segment biopsies. (A) Tumor with ill-defined boundary had the feature of infiltrative growth (hematoxylin and eosin stain, ×40). (B) Poorly differentiated cells presenting round or ovoid in shape consistent with primary nasopharyngeal carcinoma (×400). (C) Squamous eddy can be seen indicating squamous cells differentiation (×400). (D) Tumor cells were Epstein–Barr ribonucleic acid positive in situ hybridization (×400).

In the initial therapy, the patient had undergone abdominal ultrasonography for 4 times, and no hepatic lesions were noted during this period. Nevertheless, when she came to our hospital for 3-month conventional follow-up in April 2013, abdominal sonogram revealed a liver cystic lesion with thin wall and smooth margin of 18 × 16 mm in the right liver lobe, and the lesion was interpreted as a simple liver cyst (Fig. 1). Therefore, intense follow-up was suggested. Five months later, the cystic lesion enlarged to be 59 × 46 mm, with thick wall, but no signal of blood flow. Further CT presented a low density and heterogeneous lesion taking irregular wall and incomplete septa with strong contrast enhancement, indicating liver abscess (Fig. 2). However, the patient was asymptomatic, with no fever, no right up abdominal pain, and no palpable mass by physical examination. On the contrary, laboratory findings were negative, liver function was normal, the white blood cell count, C-reactive protein, and cancer-related antigen including α-fetoprotein were within the normal limits. Afterward the patient was transferred to another hospital for abscess drainage, and fluid culture was negative, but fine-needle aspiration was not performed. After drainage, the mass shrank significantly to one-third of the original size. However, it returned to previous size by CT 2 months later. Thus, the liver lesion was suspected to be malignant. Work-up examination including chest CT, pelvic MRI, emission CT for bones, gastroscopy, colonoscopy, and brain MRI excluded other lesion that may account for another primary tumor or extra-hepatic distance metastases from NPC. In addition, nasopharynx MRI showed no evidence of local relapse. On November 21, 2013, surgical resection was administrated both for histologic diagnosis and treatment, and the surgical margin was negative. Histopathologic examination definitely confirmed that the metastasis originated from NPC, since the cells of the surgical segment were similar to primary NPC on the morphology and they were positive in Epstein–Barr virus (EBV) encoded RNAs (EBERs) (Fig. 3). No adjuvant chemotherapy was done after resection of the liver metastasis.

**Figure 4:**
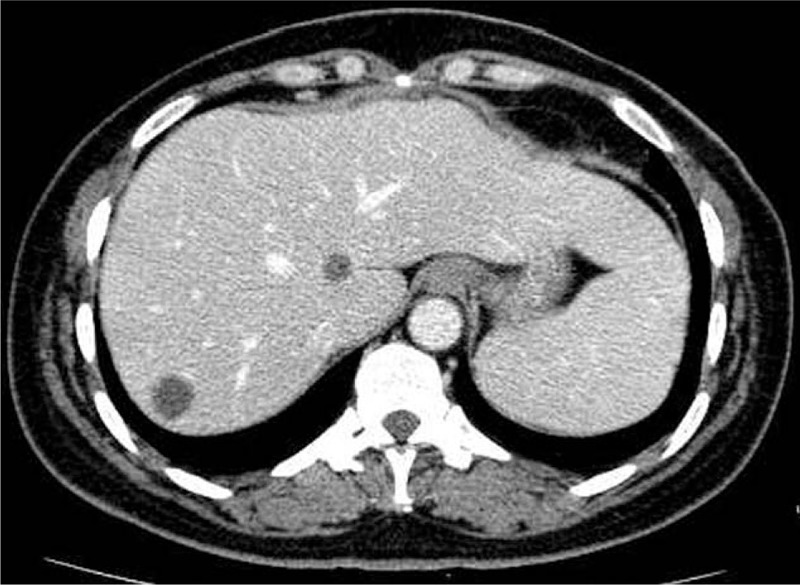
Abdominal computed tomography scan showing the recurrent small cystic lesions were extremely similar to simple cysts.

**Figure 5:**
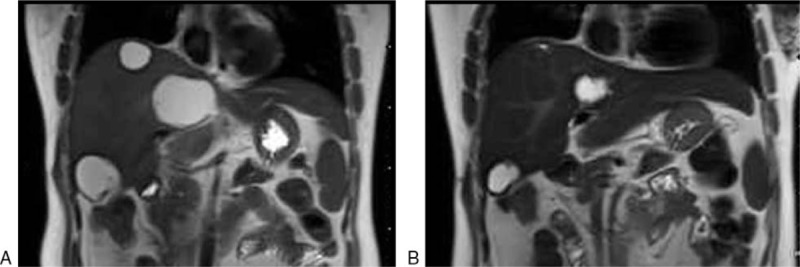
Changes of liver metastases before (A) and after (B) transcatheter hepatic artery chemoembolization treatment by abdominal magnetic resonance imaging.

However, relapse-free survival time lasted only for 8 months. In July 2014, 2 small cystic lesions were found on abdominal CT scan again, which were extremely similar to simple cysts (Fig. 4). Further contrast-enhanced ultrasonography provided no sign of “fast in and fast out,” that is, a characteristic appearance in malignant carcinoma. As a result of multidiscipline team discussion, “watch and wait” strategy was recommended. However, the number of hepatic lesions increased to 4, and the size of previous 2 cysts enlarged to be 3 cm within 3 months. The multidiscipline team members reached a consensus that the cystic lesions were metastases from NPC and suggested a palliative systematic chemotherapy. Then the patient received chemotherapy with paclitaxel and cisplatin for 6 cycles. During the first 3 cycles, partial lesions diminished in size, but stayed stable within the later 3 cycles. Nevertheless, the intraliver metastases exhibited significant progress soon after the chemotherapy. Subsequently, she underwent chemotherapy with gemcitabine plus targeted therapy with nimotuzumab, then single navelbine both for 3 cycles, but neither protocols showed notable effects on hepatic lesions. In addition, during the phase of chemotherapy with navelbine, the patient complained of chest pain, an irregular and fixed lump was found on her chest wall, approximately 3 cm in diameter. After local surgical resection was performed, the lump histologically was demonstrated to be an extra-hepatic metastasis from NPC. Considering the patient's good performance status score and grade A liver function according to the Child-pugh grading system, transcatheter hepatic artery chemoembolization (TACE) was administrated for 5 times from December 2015 to April 2016. After the TACE treatment, all the lesions were obviously decreased in size, with the largest metastasis decreasing from 64 × 53 to 33 × 32 cm (Fig. 5). Meanwhile, carbohydrate antigen 125 (CA125) decreased from 125 to 60.8 U/mL, and squamous cell carcinoma antigen decreased from 2.8 to 0.9 ng/mL. Then 4 cycles of gemcitabine plus cisplatin were offered to consolidate the clinical effects. In the next 6 months, her liver metastases maintained stable, but lung metastases were noted. In October 2016, the CT showed the liver metastases progressed. At last, she died of liver failure in March 2017.

## 3 Discussion

Cystic lesions within the liver occur up to 5% of population in clinical routine practice, covering a wide variety of pathologic entities, including benign lesions (eg, common simple cyst, parasitic cyst, Caroli disease, pyogenic abscess, echinococcal cyst), rare malignant tumors (biliary cystadenoma and cystadenocarcinoma) and malignant metastatic disease.^[3,4]^ In fact, each disease has characteristic appearance on medical imaging, the ultrasonography, CT, and MRI are usually useful tools to make differential diagnosis. But it is always difficult to make a definitive diagnosis depending on imaging method. Clinical history and laboratory data are also helpful. Further needle aspiration or an open biopsy to obtain specimens is often necessary to make a diagnosis.

It is usually easy to make the differential diagnosis between cystic liver metastases from benign hepatic cystic lesions based on imaging feature. Single cysts manifest as a well-defined homogeneous and hypodense mass, with thin wall and an absence of internal structures, when an intravenous contrast material is administrated, there is no enhancement of the wall and contents on CT imaging. Single cysts are usually asymptomatic, unless the lesions are big enough to produce pressure effect. Generally, pyogenic abscess exhibits a low-density mass and may have variable appearance on CT scans, “double target sign” which shows a central hypoattenuating lesion is surrounded by a ring of enhancing tissue and encircled by an outer rim of hypoattenuation, which is a characteristic of abscess cavity. In clinical practice, patients with liver abscess may present sepsis and space–occupying-related symptom. Most liver metastases are solid, and a small part of it is cystic. Cystic liver metastases usually come from colorectal cancer, ovarian cancer, pancreatic cancer, gastrointestinal stromal tumor, melanoma, sarcoma, neuroendocrine tumors, and so on.^[5]^ The cystic nature of metastases is due to the rapid growth beyond hepatic arterial blood supply of the lesion, leading to central necrosis. Cystic liver metastases may have heterogeneous and ill-defined borders, irregular and incomplete septa, and the inner surfaces are typically ragged with mural nodules. In addition, cystic metastases present an enhanced rim on the arterial phase of CT and MRI. 18^F^-FDG positron emission tomography-CT (PET-CT) is much more sensitive and accurate than CT and MRI. It is recommended as the preferred modality to identify malignant tumor. However, our case denied the PET-CT check because of the high costs.

The cystic hepatic lesion of the mentioned patient resembled benign single cyst with fast growth at first. After a short term, it evolved into an abscess-like mass, but finally demonstrated to be a liver metastasis from NPC. Because of the atypical feature of the liver metastasis, it took 7 months to make the diagnosis. If the oncologist realized the cystic appearance of the hepatic metastases from NPC and asked the application of biopsy earlier, the patient would live longer.

Cystic liver metastases from NPC are extremely rare. To our knowledge, there are only 3 cases reported in the published literature. One case reported by Kao et al was about a middle-aged woman diagnosed as NPC, who underwent standard local radiation therapy and chemotherapy with cisplatin and 5-flurorouracil. Only 1 year after initial therapy, multiple cystic hepatic lesions were found on sonogram and demonstrated to be liver metastases by aspiration. Though paclitaxel plus cisplatin and docetaxel were administrated, she died of live coma and failure of multiple organs in a short term after chemotherapy.^[6]^ Another report noted a 50-year-old man with a huge hematoma in the liver which was derived from a diagnosed NPC. He was treated with intermittent drainage of the liver hemotoma while receiving neoadjuvant chemotherapy and subsequent concurrent chemoradiotherapy. Then biopsy of the cystic lesion was performed, and histologic examination revealed a metastasis carcinoma from NPC, and bone metastases were detected several months later.^[7]^ The third case described a pediatric patient aged 14 with NPC metastases to liver, spleen, lymph node, and bone marrow. The liver lesions resembled benign cystic disease, but showed uptake on PET-CT. She died when receiving the 5th cycle chemotherapy.^[8]^ The prognosis of all 3 cases was extremely poor.

Because primary squamous liver carcinoma and secondary squamous hepatic metastases are similar in morphologic and immunophenotypical characters by histology, and primary squamous liver carcinoma may also reveal cystic mass on medical imaging, in fact, to make the diagnosis of liver metastases from NPC of our case, another possibility as primary squamous liver carcinoma should be ruled out, although only 30 or so cases have been reported in the literature. Primary squamous liver carcinoma has been reported to originate from pre-exist liver cysts or be associated with teratomas or liver cirrhosis.^[9]^ Our case presented no liver lesions until the primary follow-up and the relative history of liver insult was denied. Most importantly, her liver tumor resection specimen was EBERs positive in situ hybridization, highly supporting the diagnosis of a hepatic metastasis. The EBV has been proved to be strongly related to NPC, and EBER in situ hybridization is a readily available method for determining whether a tumor was metastasized from NPC.^[10]^

Almost half of the patients with NPC are at the advanced stage when the diagnosis is made, and 15% to 30% of patients in this group experience failure at distant sites.^[11]^ The most common metastatic organs are lung, bone, liver, and lymph node. With the incidence of 29.3% to 36%, liver is reported as the third most frequent metastatic site with the worst prognosis, and the median survival time is 3 to 5 months.^[12]^ The management of metastatic NPC is essentially palliative. Retrospective studies have shown this proportion of patients obtain greatly variable clinical outcomes with different ages, anatomical sites, and numbers of metastases. Therefore, individualized strategies are recommended for metastatic NPC. Not only systematic chemotherapy and aggressive local therapy but also molecular-targeted therapy can be chosen to be performed to this group of patients.

Platinum-based chemotherapy is considered to be the major systemic treatment and the most common combination of cisplatin and 5-Fu yields a response of 70% to 80%, while paclitaxel, docetaxel, gemcitabine, capecitabine combined with cisplatin achieve the similar response.^[13]^ Paclitaxel, docetaxel, gemcitabine, and capecitabine can be chosen as the second line chemotherapy regimen, achieving 30% to 40% response.^[14–16]^ However, systematic chemotherapy showed limited efforts on our patient. Epidermal growth factor receptor (EGFR) nearly expresses in all head and neck carcinoma. Anti-EGFR molecular therapy shows clinical efficiency in advanced head and neck cancer cases. NCCN guideline recommends the combination regimen as cisplatin/docetaxel/cetuximab, cisplatin/paclitaxel/cetuximab, and cisplatin/5-Fu/cetuximab for unresectable, recurrent and metastatic head and neck cancers. Nimotuzumab is a humanized IgG1 monoclonal antibody targeting EGFR. Nimotuzumab with radiotherapy has been recommended as a standard treatment for locally advanced NPC in Chinese NCCN guideline for head and neck cancers. Chinese oncologists reported that nimotuzumab plus gemcitabine treating metastatic NPC obtained objective response rate (ORR) of 61.4%,^[17]^ and the combination of nimotuzumab plus cisplatin and paclitaxel had ORR of 64.2% in the treatment of recurrent NPC cases.^[18]^ On the contrary, our patient underwent nimotuzumab plus gemcitabine and received little response.

In terms of local therapy to hepatic metastases, hepatic resection is considered to be the only local curative treatment. The role of hepatic resection has been well established in patients of colorectal and neuroendocrine carcinomas,^[19,20]^ but of noncolorectal and noneuroendocrine carcinoma patients, the effort of hepatic resection remains controversial based on limited studies and small number of patients.^[21]^ Likewise, the management of liver metastases from NPC also has not been extensively investigated. Jun Huang et al reported a comparative study of the patients with NPC with hepatic metastases who were treated by partial hepatectomy or TACE, the progression-free survival and the median overall survival of the resection group was 45.2 and 21.2 months, respectively. However, in the control group, the progression-free survival and the median overall survival were 14.1 and 4.2 months, respectively. Hepatectomy has a significant advantage over TACE.^[22]^ Other studies were sporadic case reports regarding NPC with hepatic metastases. Weitz et al reported 2 patients with NPC with hepatic metastases who underwent partial hepatectomy had a long survival time.^[23]^ Another case report was about a patient with a solitary liver metastasis from NPC treated by segmentectomy, no signs of local or distant recurrence were noted during the 6-month follow-up.^[24]^ Compared with these reports, our case only obtained a recurrence-free interval of 3 months after the partial hepatectomy. The reasons that contributing to the short recurrence-free interval may include the relatively large size of the lesion and the absence of adjuvant chemotherapy. Adam et al analyzed 1452 patients from 41 centers who received hepatic resection for noncolorectal and nonendocrine carcinoma. He proposed that squamous histology was a poor diagnosis factor, patients with head and neck cancer with squamous histology experienced poor outcome, and the 5-year survival rate was <15%.^[21]^

The TACE is a widely accepted method of palliative chemotherapy in unresectable hepatocellular carcinoma and secondary liver neoplasms. Owing to hepatic arterial supply of the metastatic lesions and targeted delivery of drug in optimal doses, TACE is less invasive over systemic chemotherapy and has been proved effective in local tumor control and the improvement of life quality. Our patient had kept a relatively long period of stable status of hepatic metastases by TACE, though the liver lesions were insufficient in blood supply. It leads to a hypothesis that cystic lesions may be sensitive to TACE which can be chosen as a preferred method for unresectable cystic liver metastases from NPC, but it still needs further evidence.

Despite systematic chemotherapy, anti-EGFR targeted therapy and local therapy were administrated. Our case experienced ex-hepatic distant metastases and hepatic metastases progression. Management of metastatic NPC is still a great challenge. In recent years, new systematic immunotherapeutic approaches for NPC are merging, like T-cell immunotherapy targeting EBV-infected carcinoma and anti-PD-1 therapy. However, these strategies remain experimental.^[11]^

## 4 Conclusion

This report presents the forth case with cystic liver metastases from NPC in the published literature. This rare cystic appearance may be a special form, which does exist for liver metastases of NPC, indicating poor prognosis. Oncologists need to enhance the recognition and diagnosis level of this type of metastases. Intense follow-up and early diagnosis are important. While emphasizing the importance of local therapy and personal principles for liver metastases, TACE may be a preferred method for unresectable cystic liver metastases from NPC.

## Author contributions

**Conceptualization:** Qiuxia Liu.

**Data curation:** Qiuxia Liu, Jianfang Wang, Caiping Sun, Jun Xu.

**Investigation:** Qiuxia Liu, Jianfang Wang, Caiping Sun, Jun Xu.

**Resources:** Qiuxia Liu.

**Supervision:** Qiuxia Liu.
